# Modeling and Visual Simulation of Bifurcation Aneurysms Using Smoothed Particle Hydrodynamics and Murray’s Law

**DOI:** 10.3390/bioengineering11121200

**Published:** 2024-11-27

**Authors:** Yong Wu, Yongjie Yan, Jiaxin Zhang, Fei Wang, Hao Cai, Zhi Xiong, Teng Zhou

**Affiliations:** 1 School of Economics, Guangdong University of Technology, Guangzhou 510520, China; wuyong@gltu.edu.cn; 2 Guangxi Key Laboratory of Culture and Tourism Smart Technology, School of Tourism Data, Guilin Tourism University, Guilin 541006, China; 3 Guangdong-Hong Kong-Macao Universities Joint Laboratory for Precision Prevention and Research of Eye Diseases, College of Mathematics and Computer Science, Shantou University, Shantou 515063, China; 22yjyan@stu.edu.cn (Y.Y.); 24jxzhang1@stu.edu.cn (J.Z.); haocai@stu.edu.cn (H.C.); zxiong@stu.edu.cn (Z.X.); 4 University of Electronic Science and Technology of China, Chengdu 611731, China; teng.zhou@hainanu.edu.cn; 5 The Hong Kong Polytechnic University, Hung Hom, Kowloon, Hong Kong 100872, China

**Keywords:** bifurcation aneurysm simulation, smoothed particle hydrodynamics, Murray’s Law, wall shear stress

## Abstract

Aneurysm modeling and simulation play an important role in many specialist areas in the field of medicine such as surgical education and training, clinical diagnosis and prediction, and treatment planning. Despite the considerable effort invested in developing computational fluid dynamics so far, visual simulation of blood flow dynamics in aneurysms, especially the under-explored aspect of bifurcation aneurysms, remains a challenging issue. To alleviate the situation, this study introduces a novel Smoothed Particle Hydrodynamics (SPH)-based method to model and visually simulate blood flow, bifurcation progression, and fluid–structure interaction. Firstly, this research consider blood in a vessel as a kind of incompressible fluid and model its flow dynamics using SPH; and secondly, to simulate bifurcation aneurysms at different progression stages including formation, growth, and rupture, this research models fluid particles by using aneurysm growth mechanism simulation in combination with vascular geometry simulation. The geometry incorporates an adjustable bifurcation structure based on Murray’s Law, and considers the interaction between blood flow, tissue fluid, and arterial wall resistance. Finally, this research discretizes the computation of wall shear stress using SPH and visualizes it in a novel particle-based representation. To examine the feasibility and validity of the proposed method, this research designed a series of numerical experiments and validation scenarios under varying test conditions and parameters. The experimental results based on numerical simulations demonstrate the effectiveness and efficiency of proposed method in modeling and simulating bifurcation aneurysm formation and growth. In addition, the results also indicate the feasibility of the proposed wall shear stress simulation and visualization scheme, which enriches the means of blood analysis.

## 1. Introduction

The proportion of people suffering from cardiovascular diseases has increased in recent years, and the high disability rate and high mortality rate of these diseases have posed significant threats to human health, attracting considerable attention from the medical and research communities [1]. Aneurysmal lesions are one of the most common diseases, with an incidence rate of 3–5% [2]. The main manifestation is a local bulge caused by arterial wall injury, and most of which occurs at the arterial bifurcation. Aiming at these pathological phenomena, this study combines fluid simulation techniques from computer graphics with the specific pathological mechanisms of blood diseases to reconstruct and simulate the progression of disease pathogenesis. This approach serves as a visual tool to assist in the clinical medical analysis, prediction, and diagnosis of diseases [3,4]. Additionally, it also has important application significance in popular science education, physiological simulation, traffic flow prediction [5], virtual technology, etc.

Among various Lagrange-based methods, Smoothed Particle Hydrodynamics (SPH) [6] has gained considerable attention thanks to its effectiveness in simulating realistic objects or scenes. Despite the success of SPH-based physical simulation in a wide spectrum of sectors, such as entertainment, manufacturing, and scientific modeling, it remains a non-trivial task to achieve favorable simulation outcomes in the medical domain. This is because developing a medical simulator, for instance, blood flow stimulation, should not only take account of the fluid aspect of blood, but also the complex physiological characteristics and behaviors. Unfortunately, this is beyond the reach of most existing fluid simulation models.

Existing studies on fluid simulation can be broadly classified into three categories: Lagrange-based methods, Euler-based methods, and hybrid methods (i.e., a combination of Lagrange- and Euler-based methods). Smoothed Particle Hydrodynamics (SPH) [6] is regarded as a typical method of Lagrange-based methods. Physical simulation based on SPH can provide realistic scene effects for film production, and can also be used as the calculation and visualization results of scientific analysis in related fields. So far, SPH-based blood simulation has some application results. Unfortunately, it remains a non-trivial task to effectively simulate blood because of the complex physiological characteristics of blood and the different fluid solid coupling behaviors in different disease scenarios, and the existing general methods can not be directly used to simulate the pathological process of vascular blood diseases.

To tackle the issues mentioned above, we analyze and integrate the medical mechanism model of aneurysmal lesions, and introduce an SPH-based simulation of bifurcation aneurysmal lesions and a visual analysis method of blood parameters. The proposed method firstly discretizes the blood into a particle model and treats blood as an incompressible fluid, and simulates it using the SPH method of density-invariant and divergence-free coupling. Secondly, combined with the physiological mechanism of aneurysm and considering the interaction of blood fluid, vessel wall proteins and tissue fluid, we solve the motion governing equation by using the properties of blood particles to simulate the fluid–solid coupling between blood and blood vessels in the growth process of aneurysm. In addition, we investigate into the under-explored modeling and simulation of bifurcation aneurysms bifurcation structure of blood vessels, and introduce a geometric model of regulated bifurcation blood vessels conforming to Murray’s Law in order to reflect the generalization of the method. In addition, we discretize the wall shear stress calculation by SPH method and provide a visual analysis method based on particle form to assist medical analysis.

The main contributions of this research are as follows: (1) we introduce a novel method to transform the pathological mechanism of aneurysm into a computational model using SPH; (2) we propose SPH-based simulation method for bifurcation aneurysms based on the bifurcation vessel scenario that satisfies the vascular structure law; and (3) we present a new visualization system that allows users to flexibly adjust relevant parameters associated with blow flow and aneurysms, and this will meet the diverse needs of various medical applications with regard to simulation, exploration, and visual analysis of the formation and progress of aneurysms in different simulation settings. The experimental results show that the proposed model can effectively simulate blood flow and aneurysm formation.

## 2. Related Work

We begin with a brief review of SPH-based fluid simulation and its application on blood simulation, followed by the relevant studies about mechanism of aneurysm lesion. In either aspect, the existing methods demonstrate insufficient flexibility and richness in aneurysm simulation.

### 2.1. SPH-Based Fluid Simulation

SPH is one of the most widely used Lagrangian methods in fluid simulation. It has been continuously updated and improved since Muller et al. [6] firstly applied it to the Navier–Stokes equation to implement fluid simulation. Even though SPH was originally designed for compressible fluid, it has now been developed and used to simulate incompressible fluid (e.g., water, blood) [7,8,9,10,11,12,13]. These existing methods strive for a larger time step and larger scale simulation, ensuring stability in less time consumption.

Recently, divergence-free SPH (DFSPH), proposed by Bender et al. [12,14], successfully meets the incompressibility condition of density-invariant and divergence-free step by step, which is achieved by solving the stiffness coefficient. Due to the advantage in efficiency and stability, DFSPH has been widely adopted by many fluid simulators. Furthermore, Wang et al. [13,15] proposed the Coupling Incompressible SPH (CISPH) method, which takes the particle displacement as the correction variable according to Position Based Fluid (PBF) [9] and couples the incompressible fluid conditions differing from DFSPH, successfully reducing the cost of computation and ensuring the stability of fluid simulation. Considering the similar stability with less time consumption comparing from DFSPH, we develop our simulation method based on CISPH, namely the scheme coupling density-invariant and divergence-free.

### 2.2. Blood Simulation Application

In the application of blood simulations, the finite element simulation method is widely used, but this research mainly focuses on SPH method, and the application of SPH method in blood simulation. For SPH-based blood modeling, Chen et al. [16] proposed a real-time blood flow simulation method based on shape constraints, ensuring the viscous effect of blood simulation and accelerating the simulation speed simultaneously. Shahriari et al. [17] applied SPH method to the modeling of cardiovascular flow and simulated unstable blood flow in the cardiovascular system. More recently, Topalovic et al. [18] modeled blood vessel wall and blood inside as FEM shell elements and SPH particles, respectively, and used nodes to surface algorithm to contact between them to investigate the means of blood flow modeling. Similarly, Topalovic et al. [19] further facilitated SPH method to simulate blood in complex geometrical vessels, and proposed a lifecycle algorithm to reuse blood particles during the simulation. Focusing on the fluid–solid coupling behavior between blood and substances in blood vessels, Chen et al. [20] proposed a thrombus formation simulation method based on Gillespie’s method, which integrates the blood medical model into the SPH method, and simulates the process of blood coagulation and thrombus formation after vascular trauma by using the physicochemical reaction of thrombus formation. On this basis, Wang et al. [21] introduced the velocity decay factor to further improve the simulation method of thrombus formation, making the fluid solid coupling simulation effect between blood and thrombus more realistic. For combining blood simulation with aneurysms, Leonardo et al. [22] used SPH method for numerical simulations of blood flow through a brain vascular aneurysm with an adjustable artificial stent to analyze impacts on blood changes in aneurysms. Costanza et al. [23] developed a specific tool to estimate the blood flow-induced loads and test in a vessel with an abdominal aorta aneurysm. However, due to the complexities lie in physiological characteristics of blood and differences in different environments, the realization of real blood flow simulation requires modeling from the specific physiological mechanism of blood.

### 2.3. Mechanism of Aneurysm Lesion

In terms of the mechanism of aneurysm lesion, Perkold et al. [24] analyzed the saccular aneurysm of bifurcated vessels by numerical method, and simulated the blood flow after aneurysm lesion in the form of blood particles based on hemodynamics. However, this simulation is limited to the two-dimensional plane, which has difficulty meeting the demand. Hademenos et al. [25] constructed a nonlinear numerical model of aneurysm growth and rupture according to the medical analysis of aneurysms, and analyzed the difference between the results of different models and clinical data through experiments. Nikolov et al. [26] presented a similar work, in which they constructed a biomathematics model of the blood flow in the aneurysm, and further analyzed the relationship between the aneurysm diameter and the risk of growth and rupture. From the perspective of biophysics, Badgaish et al. [27] considered the interaction between cerebrospinal fluid and blood on the wall of diseased blood vessels, combined with the comprehensive analysis of vascular proteins and other substances, and proposed a nonlinear dynamic analysis method for the growth simulation of aneurysms. In addition, Texakalidis et al. [28] reviewed previous studies on the formation, growth, and rupture of aneurysms from the biological and physical perspectives. However, most of the existing aneurysm studies are based on two-dimensional plane simulation or static simulation of aneurysms, and their analysis methods rarely involve SPH. Additionally, blood flow parameter analysis in blood simulation can provide important support for medical diagnosis.

## 3. Proposed Method

The proposed computational method for simulation and visualization of bifurcation aneurysms is mainly composed of three parts: simulation of aneurysmal lesion based on SPH, discretized computation of Wall Shear Stress (WSS) in particle form, and geometric model construction of bifurcating artery based on Murray’s Law. In the following sections, we will firstly describe the SPH method for incompressible fluid simulation, and then elaborate on the aneurysmal lesion model, computation of WSS and bifurcating artery model, respectively.

### 3.1. SPH for Incompressible Fluids

For the simulation of incompressible fluids such as blood, SPH discretizes the fluid into particles, and uses kernel functions for numerical approximations of physical attributes. Additionally, it satisfies the incompressible condition of the fluid by correction of density error and divergence-free velocity field based on continuity equation.

Generally, fluid obeys the Navier–Stokes equation and continuity equation, i.e., the law of conservation of mass, which can be expressed as

(1) $$\rho \frac{Dv}{Dt}=-\nabla p+\mu \nabla^{2}v+F^{adv}$$

and

(2) $$\frac{\partial \rho }{\partial t}+\rho \nabla \cdot v=0$$

respectively, where ρ is the density of particles, **v** is the particle velocity, $\frac{Dv}{Dt}$ is the acceleration of particles, μ is viscosity coefficient, and ∇ and $\nabla^{2}$ are the Hamiltonian operator and Laplace operator, respectively. In total, $F^{adv}$ is the additional force, and −∇p and $\mu \nabla^{2}v$ are the pressure and viscosity force, respectively.

To calculate physical attributes (e.g., density, pressure, viscosity force) of a common particle *i*, the kernel method is used, and the discrete computing method is as follows:

(3) $$A_{i}=\sum_{j}A_{j}\frac{m_{j}}{\rho_{j}}W\left(r_{i}-r_{j},h\right)$$

where $A_{i}$ represents one physical attribute of particle *i*, $r_{i}$ and $r_{j}$ are the positions of particle *i* and its neighbors, respectively, $m_{j}$ is mass of neighboring particles, W(·) is a kernel function, and *h* is the effective radius of the kernel.

In the study of the Smoothed Particle Hydrodynamics (SPH) method, smoothing kernel functions are defined, with commonly used smoothing kernels in three-dimensional space being $W_{poly6}\left(x,h\right)$, $W_{spiky}\left(x,h\right)$, and $W_{viscosity}\left(x,h\right)$, which are used in the calculations of density, pressure, and viscosity forces, respectively. The calculations of pressure and viscosity forces require the corresponding first and second derivatives, i.e., $\nabla W_{spiky}\left(x,h\right)$ and $\nabla^{2}W_{viscosity}\left(x,h\right)$. The expressions for the respective smoothing kernels and their derivatives are as follows:

(4) $$W_{poly6}\left(x,h\right)=\frac{315}{64\pi h^{9}}\left\{\begin{array}{cc} {\left(h^{2}-x^{2}\right)}^{3}, & 0\leq x\leq h\\ 0, & \mathrm{otherwise} \end{array}\right.$$

(5) $$\nabla W_{spiky}\left(x,h\right)=-\frac{45}{\pi h^{6}x}\left\{\begin{array}{cc} {\left(h-x\right)}^{2}x, & 0\leq x\leq h\\ 0, & \mathrm{otherwise} \end{array}\right.$$

(6) $$\nabla^{2}W_{viscosity}\left(x,h\right)=\frac{45}{\pi h^{6}}\left\{\begin{array}{cc} (h-x), & 0\leq x\leq h\\ 0, & \mathrm{otherwise} \end{array}\right.$$

In this way, the Navier–Stokes equation (Equation (1)) can be solved, and the position and velocity of particles at each frame can be updated.

Since blood is regarded as an incompressible fluid, it is not enough for the blood simulation, and therefore, we further focus on incompressibility. Simulation of incompressible fluid, as it is known, needs to satisfy the following incompressible conditions, i.e., density-invariant and divergence-free:

(7) $$\frac{\partial \rho }{\partial t}=0\Leftrightarrow \nabla \cdot v=0$$

However, the SPH method needs further modification to meet these conditions, as a consequence of being designed for compressible fluids originally. At present, a considerable number of SPH methods have been improved for the incompressibility, among which CISPH, recently proposed by Wang et al. [13,15], has performed well in terms of efficiency and effectiveness because of the coupling of density-invariant and divergence-free conditions in the solution. In this research, we thus adopt the correction method of CISPH in our simulation of blood flow, and the details of CISPH are introduced in [15].

### 3.2. Simulation of Aneurysmal Lesion

Since the blood flow and tissue fluid, as well as the collagen and elastin in the artery act as vital roles on the deformation of arterial wall, it is important to combining them with the force analysis of arterial wall to construct a reliable simulation model of aneurysmal lesion. In the process of lesion, we focus on the growth of aneurysm, i.e., the increase in size of aneurysm. Inspired by [27], a novel mathematical and computational model of aneurysmal lesion combined with SPH is proposed, which consists of the interaction between blood, tissue fluid and arterial wall and the increase in aneurysms.

In our simulation model, we firstly consider the force that causes the aneurysm to expand outward $F^{aneurysm}$, which combines the forces of blood flow and tissue fluid flow acting on the arterial wall and the resistance of collagen and elastin in the arterial wall, given by

(8) $$F^{aneurysm}=F^{blood}-F^{fluid}-F^{protein}$$

For the force of blood flow $F^{blood}$, we compute it by Navier–Stokes equation (Equation (1)) combined with the impulse of blood on the arterial wall, i.e., Ft=mv, which can be expressed as

(9) $$F^{blood}=\frac{m}{\rho }\left(-\nabla p+\mu \nabla^{2}v+F^{adv}\right)$$

As blood exists in the form of particles in SPH method, the maximum impact force of all particles involved in the computation is taken as the approximate force of blood fluid acting on the arterial wall, which is expressed as

(10) $$F^{blood}=\max_{i\in S}\frac{m_{i}}{\rho_{i}}\left(-\nabla p_{i}+\mu \nabla^{2}v_{i}+F_{i}^{adv}\right)$$

where *S* is the collection of particles involved in the computation of force of blood acting on arterial wall. In this way, $F^{blood}$ can be solved by converting the computation of $\rho_{i}$, $-\nabla p_{i}$ and $\mu \nabla^{2}v_{i}$ into SPH form based on Equation (3).

For the force of tissue fluid flow $F^{fluid}$, it is assumed to be a pseudo fluid, and is obtained directly from a biomechanical method [27] in a simplified way without using SPH, to reduce the complexity of our computational model. Firstly, the tissue fluid is considered compressible, and then we formulate $F^{fluid}$ employing a simplified model of compressible inviscid Euler equation [27], which is expressed by the product of pressure and the cross-sectional area *s* acting on the aneurysm. By variable replacement and simplification, $F^{fluid}$ is expressed as:

(11) $$F^{fluid}=\left(\rho_{f}cs\right)v_{w}$$

where $\rho_{f}$ and *c* are the density of tissue fluid and the propagation speed of wave equation, respectively, and $v_{w}$ is the lesion velocity.

For the resistance of collagen and elastin $F^{protein}$, we model it using a simple yet effective spring mass system where the $F^{protein}$ is related to the cross-sectional area *S* and stress σ of collagen and elastin,

(12) $$F^{protein}=\sum_{i\in \{C,E\}}K_{i}S_{i}\sigma_{i}$$

where *K* is the scale factor, and collagen and elastin are represented by subscript *C* and *E*, respectively. For the stress in Equation (12), we represent it as σ=ϵ according to the linear relationship between stress and strain. Additionally, the relation between strain and radius *R* of aneurysm from [25] is further given by $\epsilon =\frac{\Delta R}{R}$, where ΔR is the increment of *R*. Thus, the stress can be expressed as

(13) $$\sigma =\frac{\Delta R}{R}$$

Combining these relations with Equations (8)–(13) with the calculation of velocity and acceleration in dynamics, the expression of increment of aneurysm radius ΔR is converted as

(14) $$\Delta R=F^{blood}/\left(\frac{m}{t^{2}}+\frac{\rho_{f}cs}{t}+\sum_{i\in \{C,E\}}\frac{K_{i}S_{i}}{R}\right)$$

As a result, the physical changes in the arterial wall have been incorporated into the simulation of blood using SPH method, and the process of aneurysmal lesion can be restored by the graphics method.

In comparison to the previous work by Badgaish et al. [27], who employed a spring-mass system to model the interaction between the arterial wall and blood flow, and used a simplified Euler equation to model the surrounding cerebrospinal fluid (CSF), our approach offers significant advancements. Although this model provides insights into aneurysm growth and rupture prediction, it does not fully capture the fluid–structure interactions within a particle-based framework. In contrast, we have adopted the Smoothed Particle Hydrodynamics (SPH) method, which is better suited to simulate the behavior of incompressible fluids, especially in complex biomechanical environments. Furthermore, we have incorporated SPH modifications that ensure density invariance and divergence-free conditions, which not only enhance the accuracy of the simulation, but also optimize computational efficiency.

### 3.3. Computational Method of WSS Based on SPH

WSS is one of the most important dynamic parameters in the study of blood flow, which can provide indicators for evaluating the risk of diseases, such as aneurysms, atherosclerosis, etc. Meanwhile, taking the fact that WSS can be simply calculated by finite difference method while is not suitable for particle-based methods, e.g., SPH, we discretize the computation of WSS using SPH method tentatively, to obtain an effective computational method for visual analysis of blood system with diseases.

According to [29,30], WSS is defined as the tangential force per unit area exerted by the fluid flow on the catheter surface, which can be understood as the friction between blood flow and arterial wall in blood simulation. At a point *i* on the arterial wall, the continuous WSS is expressed as

(15) $${WSS}_{i}=-\mu \frac{dv_{t}}{dx_{n}}$$

where μ is the blood viscosity, $v_{t}$ is the blood flow velocity tangent to the arterial wall, $x_{n}$ is the normal coordinate perpendicular to the arterial wall, and $\frac{dv_{t}}{dx_{n}}$ represents the gradient of the tangential velocity on arterial wall relative to the normal coordinate of the wall, known as shear rate. The unit of WSS is Pascal (Pa).

Referring to the common formula of SPH (Equation (3)), it can be found that the discretized computational method of WSS is given by

(16) $${WSS}_{i}=-\mu \sum_{j}m_{j}\frac{v_{t,j}}{\rho_{j}}\nabla_{n}W\left(x_{i}-x_{j},h\right)$$

where $\nabla_{n}=\frac{x_{n,i}-x_{n,j}}{|x_{i}-x_{j}|}\frac{\partial }{\partial x}$, which represents the gradient in the $x_{n}$ direction from particle *i* to *j* in kernel method. Additionally, WSS on the arterial wall is expressed by the blood fluid particle located on the wall. In this way, we take the particles into computation whose distance from arterial wall is less than a certain threshold.

**Figure 1:**
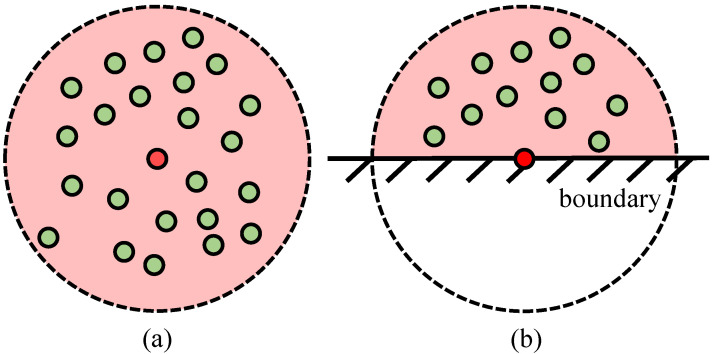
Neighboring particles of boundary particle and non-boundary particle. Red and green particles represent the central particle and neighboring particles, respectively. (**a**) is the non-boundary particle with its neighbors, while (**b**) is the boundary particle.

As a matter of fact, only the neighboring particles within the kernel radius will contribute to the calculation in kernel method of SPH. Unfortunately, the particles representing the arterial wall in WSS computation are at the boundary of the fluid domain, as shown in Figure 1. In this case, it causes a certain deviation in the sum calculation of WSS, because of the limited number of neighboring particles.

To tackle this issue, Shahriari et al. [17] named it kernel truncation, and introduced a correction coefficient to ensure the correctness of the calculation without modifying the kernel functions. In this research, we construct a more explicit computational method of WSS based on the correction coefficient.

Considering that the number of particles within the kernel radius in SPH method is generally maintained in a certain range, the computation of correction coefficient of each particle $C_{\xi ,i}$ takes the average number of neighbors of all particles as the standard, which is given by

(17) $$C_{\xi ,i}=\frac{\bar{N}}{N_{i}}$$

where $\bar{N},N_{i}$ are the average of neighbors and the current neighbors number of particle *i*, real-time calculations are performed for each frame in the visualization. Hence, the discretized computation of WSS with correction coefficient can be expressed as

(18) $${WSS}_{i}=-\mu \sum_{j}m_{j}C_{\xi ,i}\frac{v_{t,j}}{\rho_{j}}\nabla_{n}W\left(x_{i}-x_{j},h\right)$$

Eventually, it can be monitored and visualized to dynamically analyzed the characteristics of WSS through this SPH computational method. Meanwhile, it can be extended to the computation and visualization of other blood parameters.

When compared with the studies of Kageyama et al. [31] and Koutsiaris [32], our WSS calculation method shows certain similarities with their experimental results. Kageyama et al. used high-resolution imaging techniques to measure WSS at arterial bifurcations in the cardiovascular system, observing higher WSS values in these regions, particularly at the branching arteries. Koutsiaris focused on WSS measurements in the ocular microcirculation using Particle Image Velocimetry (PIV) to obtain WSS data from small vessels. Although our study does not involve direct measurements, we simulate blood flow dynamics and visualize WSS using the SPH method. The simulation results show trends in WSS that are consistent with those observed in the aforementioned studies, particularly in regions of high shear stress. This highlights the consistency of flow dynamics across different methods and models, with our SPH-based approach offering more intuitive blood flow visualization. Ultimately, the SPH method provides a robust tool for both the simulation and visualization of hemodynamic parameters, offering insights into the dynamics of blood flow and potential applications in the study of vascular diseases.

### 3.4. Simulation of Blood Flow in Murray’s Law-Based Bifurcations

Since the occurrence of aneurysm is common in bifurcating artery, and it is value to restore aneurysmal lesion in arteries with complex structures, we focus on the bifurcating artery to as blood flow environment. In order to reflect the diversity of simulation, a parameter-adjustable geometric model of bifurcating artery is constructed in this research. In detail, the shape and scale of bifurcating artery is determined according to Murray’s Law [33,34], which has been shown to be a good approximation for the cardiovascular system.

According to Murray’s Law, the relation of radius between parent and daughter vessels in the general bifurcating vessel is given by

(19) $$r_{p}^{n}=\sum_{i=1}^{N}r_{d_{i}}^{n}$$

where $r_{p},r_{d_{i}}$ are the radius of parent and i-th daughter vessel, respectively. *N* is the sum of daughter vessels whose value is 2 in our model, and *n* is the bifurcation index of vessels which value is 3 under the optimal structure. Based on the formula of Murray’s Law (Equation (19)), we parameterize the structure of the bifurcating vessel and 3 variables are taken as the adjustable parameters in our geometric model, which consists of bifurcation index (*n*), bifurcation angle (*A*), and bifurcation radius ratio (*r*), where $r=\frac{r_{d_{1}}^{3}}{r_{d_{2}}^{3}}$.

For the bifurcation angle (*A*), α and β are used to represent the two bifurcations, respectively. Based on the work in [33], it embodies Poiseuille’s law of flow to obtain a simple deduction of relation between energy and radius, and further obtain the expression of bifurcation angle by the radius of arteries,

(20) $$\begin{array}{cc} \cos(\alpha )= & \frac{r_{p}^{4}+r_{d_{1}}^{4}-r_{d_{2}}^{4}}{2r_{p}^{2}r_{d_{1}}^{2}};\;\;\cos\left(\beta \right)=\frac{r_{p}^{4}+r_{d_{2}}^{4}-r_{d_{1}}^{4}}{2r_{p}^{2}r_{d_{2}}^{2}}\\ \; & \cos\left(A\right)=\frac{r_{p}^{4}-r_{d_{1}}^{4}-r_{d_{2}}^{4}}{2r_{d_{1}}^{2}r_{d_{2}}^{2}} \end{array}$$

where A=α+β. Converting Equation (20) by arccos function to obtain the value of bifurcation angles, and combining it with Equation (19), the axis and radius of arteries can be determined, and the bifurcating arteries of different structures can be further expressed.

Furthermore, instead of using triangle patches or particles to represent arterial boundary, our bifurcating artery is expressed as an implicit geometry, which can be more flexible to represent the shape and scale of bifurcating artery and greatly reduce computational and spatial complexity. To make sure the tightness of arteries at the bifurcation, we smooth the vessel appropriately based on a smoothed surface function in mathematical form, where the surface of boundary projected onto a 2D plane can be expressed as follows:

(21) $$F\left(d\right)=a_{1}G\left(a_{2}d+b_{2}\right)+b_{2}$$

where *d* is a distance of the junction area of parent and daughter vessels, $a_{1},a_{2},b_{1},b_{2}$ are the coefficients of linear functions, and $G\left(x\right)=\frac{1}{1+\exp\left(-x\right)}$ is a nonlinear function here, which is easy to deduce the normal vector on the junction site by derivative. Additionally, in order to achieve the smoothness, it should be continuous at the junction, i.e., the first order derivative $\frac{dF}{dd}{|}_{d_{junction}}=0$, where the derivatives at parent and daughter vessels are 0.

By setting the relations mentioned above in the initialization before simulation, and applying the general boundary detection and collision handling on bifurcating artery, it finally can assist in the simulation of blood flow and aneurysmal lesion as a boundary of fluid flow domain.

### 3.5. Experimental Setup and Rendering Methods

To evaluate the effectiveness and efficiency of the proposed method, we implemented the proposed method in C++ with Microsoft Visual Studio 2015 as the integrated development environment. All of the experiments were carried out on a PC running Windows 10 as its operating system and configured with an Intel Core i7 8565U @ 1.8 GHz processor with 8GB RAM. In addition, we use OpenGL 4.5 and Blender 3.6.10, two open-source 3D creation tools, for real-time and offline rendering, respectively. Due to the realization of particle circulation treatment, the process of aneurysm lesions can be better restored with fewer particles. Therefore, the number of blood particles used in this experiment is 13,572. In addition, the basic physical parameters of blood fluid and parameters of aneurysm growth model in initialization are shown in Table 1.

The fluid rendering engine adopts a client–server architecture, and is divided into two modules: the rendering pipeline module and the fluid simulation module. The fluid simulation module is responsible for implementing the Smoothed Particle Hydrodynamics (SPH) method, and calculating the fluid particle positions for the next time step. It then injects the particle position data into the rendering pipeline, which progressively renders the scene and the screen-space fluid. Once rendering is completed, the image frame is displayed directly on the screen, after which the process returns to the fluid simulation module to continue stepping, achieving real-time fluid animation.

The rendering pipeline module is implemented using OpenGL and OpenGL Shading Language (GLSL). It employs a specialized rendering pipeline for screen-space fluid rendering techniques that process the generated images for fluid particles. This design includes vertex processing, triangle processing, rasterization, fragment shading, and framebuffer operations, with particular emphasis on generating depth textures and thickness textures, applying bilateral Gaussian filtering for smoothness, and implementing physics-based reflection and refraction effects to ensure efficiency and realism in fluid rendering.

The fluid simulation module focuses on leveraging the parallel computing capabilities of the GPU to accelerate fluid solving and neighbor particle searches, using CUDA C++ for implementation. This effectively enhances the computational efficiency of the fluid simulation, allowing the engine to maintain around 20 fps when handling fluid simulations at a scale of 100 k particles, thus enabling smooth fluid rendering.

## 4. Results and Discussion

### 4.1. Bifurcated Blood Vessel Simulation

In view of the structural complexity of bifurcated vessels, this research analyzes the influence of bifurcated vessels on hemodynamics of left coronary artery based on [35], and uses the control variable method to design three groups of blood simulation experiments under the vascular model with different bifurcated vessel parameters, so as to illustrate the feasibility and effectiveness of the vascular model with adjustable bifurcated structure in this research. Table 2 shows the design scheme of adjustable vascular parameters used in the experiment in this research. According to Murray’s Law, the bifurcation angle is $75^{\circ }$ and the bifurcation exponent is 3, which is the optimal structure of bifurcation vessels. Therefore, in the scheme design, structural parameters with bifurcation angle A of $75.00^{\circ }$, bifurcation index N of 3, and radius ratio r of 1.0 was taken as the reference group, namely, schemes G2, G5, and G9 in Table 2.

**Figure 2:**
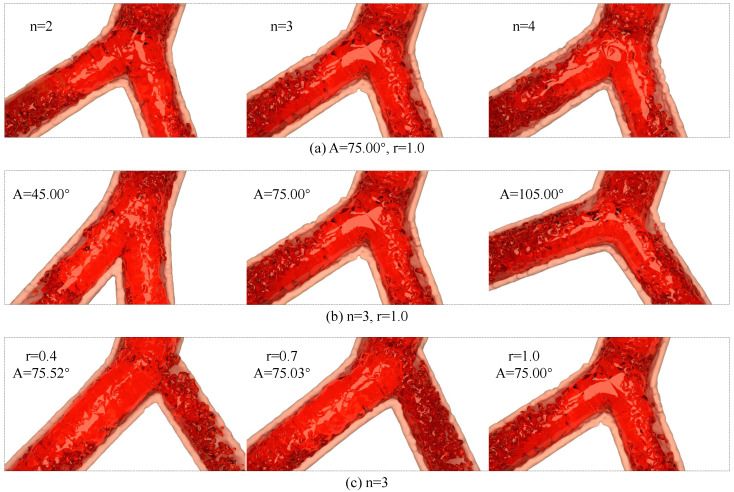
Blood simulation under bifurcating arteries with different structures. Rendering results consist of all cases in Table 2, where (**a**) corresponds to G1–G3, and (**b**,**c**) are G4–G6 and G7–G9, respectively.

Figure 2 shows the blood simulation effect under different bifurcation vessel parameters. The blood and blood vessel walls are rendered with translucent red material and pink yellow material, respectively. Figure 2a–c correspond to the schemes in Table 2, respectively. From the rendering results, the proposed method can simulate blood flow under different bifurcation structures, and obtain more realistic results.

**Figure 3:**
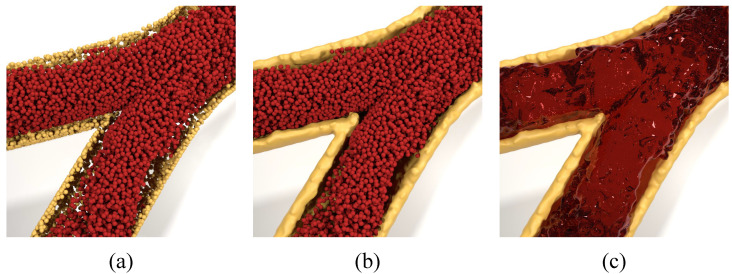
Particle form and mesh form of blood and artery. (**a**) is rendering result of all in particle form, (**b**) is rendered as the particle form for blood with mesh for the artery, and (**c**) is rendering result of all in mesh form.

In order to more intuitively illustrate the blood simulation based on SPH pure particle method in this research, Figure 3 shows the rendering results of blood and blood vessel walls in two different forms: (a) where both blood and blood vessel walls are represented as particles, (b) where blood is represented as particles and blood vessel walls as a grid fluid, and (c) where both are represented as grid fluids. To facilitate the observation of blood flow patterns, the top part of the blood vessels is hidden in the experimental rendering. The blood flow inlet end of the blood vessel is in the upper part of the vessel in the figure.

### 4.2. Simulation of Bifurcation Vessel Aneurysm Lesion

**Figure 4:**
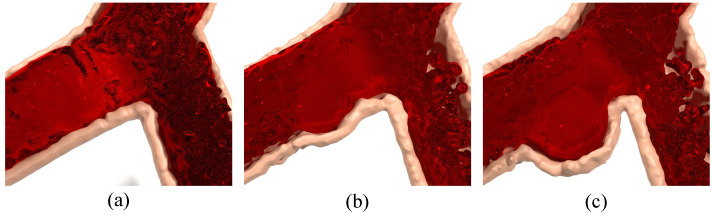
The simulation of aneurysmal lesion under bifurcating artery. (**a**) is the normal state before lesion, and (**b**,**c**) are blood flow with aneurysm in bifurcating artery, where (**c**) is after (**b**).

Aneurysm lesion is a relatively long process, and the growth of aneurysm lesion was accelerated in the simulation experiment in this research. Because the shape of aneurysms is different, but they tend to be spherical, the aneurysms in this experiment are simulated to be cystic. Figure 4 shows the simulation process of the aneurysm lesion from the normal state of (a) to the initial bulging of (b), and finally to the complete development of (c). Corresponding to the specific simulation (a), (b), and (c) in Figure 4 are the 900th, 3000th, and 6000th frames, respectively.

**Figure 5:**
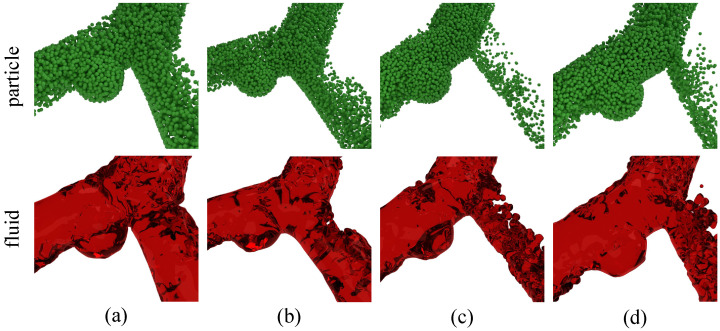
Aneurysms developed to a certain extent under bifurcating arteries. (**a**–**d**) are the rendering results of aneurysm corresponding to G1, G6, G7, and G8 in Table 2, respectively.

In order to prove the universality of the aneurysm lesion simulation proposed in this research, the experiment of the bifurcation aneurysm lesion simulation was carried out on the bifurcation vessels with different structures. To realize aneurysm simulation under different vascular structures, only the aneurysm lesion site needs to be set before the beginning of simulation. Figure 5 shows the simulation results at the later stage of aneurysm growth (frame 5000) under four different bifurcation vessel structures with obvious discrimination. In order to observe the blood morphology more clearly, only the blood particle morphology and grid liquid morphology are presented in the figure, and the blood vessel model is hidden. Columns (a), (b), (c), and (d) in Figure 5 correspond to schemes G1, G6, G7, and G8 in Table 2, respectively. The first is a blood particle made of green material, and the second is a grid liquid made of translucent red material.

The experimental results show that the proposed method can effectively simulate aneurysm lesions, and has good generalization ability and simulation authenticity.

### 4.3. Visual Analysis of Blood Flow Parameters

**Figure 6:**
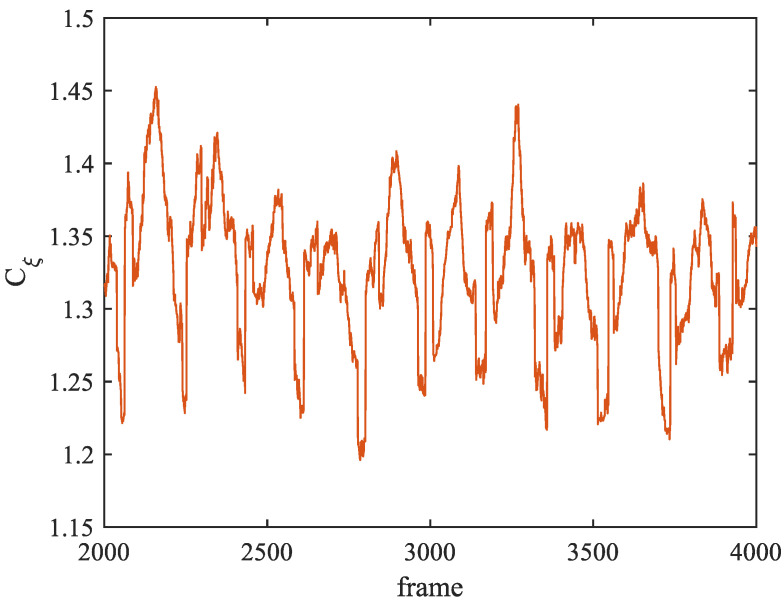
Average change in $C_{\xi }$ from 2001st frame to 4000th frame.

**Figure 7:**
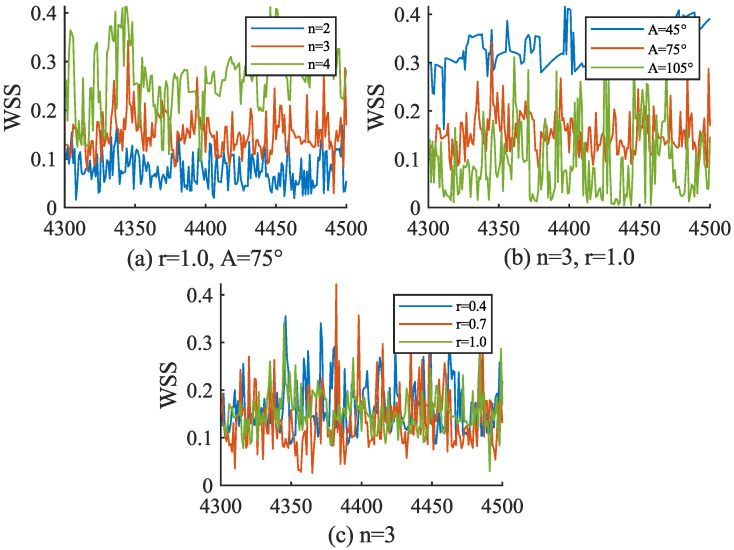
Changes in WSS at the orifice of the aneurysm. WSS is measured in pascals (Pa). (**a**–**c**) are the WSS curves corresponding to G1–G9 in Table 2 from 4300th frame to 4500th frame.

As a crucial blood flow parameter, WSS is discretized by the SPH method in this research. According to the calculation of $C_{\xi }$ in Equation (17), the number of neighboring particles has a strong correlation with the calculation result of WSS, so the experiment analyzes the change in $C_{\xi }$. Figure 6 shows the mean change in $C_{\xi }$ corresponding to all blood particles participating in the WSS calculation as vessel walls in each frame from frame 2000 to frame 4000 of the simulation. It can be found from Figure 6 that the value of $C_{\xi }$ is maintained between 1.20 and 1.45, and presents obvious periodic changes. It shows that the calculation method of WSS in this research is feasible. The periodic change shown in the figure is caused by particle circulation, which also indicates that the experimental blood simulation can restore the pulse of the blood system to a certain extent. Since WSS can reflect the characteristics of vascular diseases, this experiment carried out a visual analysis of WSS at the aneurysm mouth. Figure 7 shows the WSS curve from frame 4300 to frame 4500 at the aneurysm mouth under the bifurcation vessel structure scheme listed in Table 2. Figure 7a,b show the WSS changes at the tumor mouth under different vascular structures with N and A as variables, respectively. It can be found from the curves in the figure that the WSS values of different bifurcation index N and different bifurcation Angle A have obvious differentiation, while Figure 7c takes the radius ratio as the variable value curve, it can be found that there is no significant difference in WSS changes under different r values. According to Murray’s Law, the vascular structure (G7, G8, G9) in Figure 7c is close to the optimal structure, so the small difference in the figure reflects the reliability of the WSS calculation method in this research. As for Figure 7a,b, the deviation of bifurcation index and bifurcation angle both have an impact on the size of WSS. Therefore, monitoring the size of WSS at the aneurysm orificium in the simulation can be used as an indicator for further analysis of aneurysm lesions.

**Figure 8:**
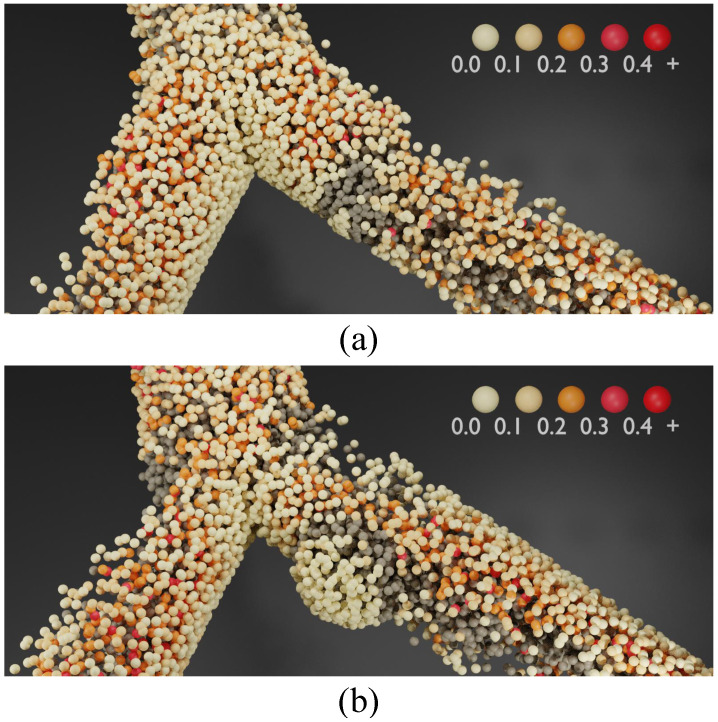
Visualization of WSS in particle form. Colors of particles are grading by the value of WSS from canary yellow to red, while non arterial wall particles are colorized as gray. (**a**,**b**) are WSS in bifurcating artery without aneurysm and with aneurysm, respectively. Note that the particles in the center of the artery are involved in the WSS calculation, and their colors can be interpreted as representing the magnitude of fluid-induced shear stress.

In order to further analyze the visualization of WSS in blood vessel walls in the blood simulation, the experiments in this research present WSS in the form of three-dimensional particles, and as a visualization method for the SPH method to enrich the analysis method of WSS. Figure 8 shows the visualization results of blood flow WSS in (a) and (b) without aneurysm and with aneurysm. In the figure, the blood particles involved in WSS calculation are also regarded as wall particles, and the size of WSS is distinguished according to the color level. The lighter (light yellow) particles represent the smaller WSS at the site, and the darker (red) particles represent the larger WSS, while the remaining particles are pure blood particles, represented by gray material. Observing the color distribution of particles, we can see that the WSS visualization method presented in the form of particles in this research can effectively reflect the distribution of WSS.Furthermore, we provide a demonstration video to showcase the visualization effect, which is available in the (Supplemental Video S1).

### 4.4. Efficiency Evaluation of the Proposed Method

The proposed simulation method is based on the SPH method coupled with the hematology model. In our implementation, we use CUDA parallel computation to speed up the simulation process. In order to verify the real-time performance of our method, we designed four groups of bifurcation blood flow simulation experiments under different test conditions (TC) to investigate the running time of the algorithm and the impact of particle number and aneurysm lesion on simulation efficiency.

Table 3 shows the experimental results obtained with the four test conditions. In this experiment, the number of frames per second (FPS) is used as a metric for efficiency evaluation. As can been seen from the table, the proposed method achieves a FPS of 86.95 by using CUDA under the TC1, in which a total number of 5520 particles was involved in the simulation. With the increase in the number of particles, the proposed method shows a reasonable decrease in simulation efficiency, achieving a FPS of 54.56 with a number of 15,456 particles and a FPS of 11.10 with a number of 40,296 particles by using CUDA under the TC2 and TC4, respectively. These results demonstrate that the proposed method can be used to simulate aneurysms with a moderate number of particles in a real-time fashion. In our experiments, we found that a range of the number of particles from 10,000 to 20,000 can achieve a good balance between the simulation quality and efficiency. In addition, by comparing TC2 and TC3, we can also observe that there is little impact on the efficiency when simulating blood flow in artery with aneurysm lesion.

## 5. Conclusions

This study combines the geometric bifurcated artery model, which adheres to Murray’s law, with an aneurysm growth model. The wall shear stress (WSS) and blood flow parameters are computed using SPH discretization, simulating the fluid–structure interaction between blood flow and vascular walls during aneurysm growth. Additionally, a parameter-adjustable bifurcated vascular geometric model is constructed to simulate blood flow and aneurysm growth in bifurcated vessels. The simulation results are visualized through graphical methods, providing a visual reconstruction of the aneurysm growth process. Murray’s law is fully utilized to achieve diversity in bifurcated vascular structures.

The feasibility and effectiveness of the proposed method are further demonstrated through simulation experiments on blood flow and aneurysm growth in bifurcated vessels. The results show that the method effectively visualizes the aneurysm growth process and discretizes the blood parameter WSS using the SPH framework in particle form. The proposed method produces high-quality visual effects and provides effective visual support for related fields.

Due to the SPH method’s discrete representation of blood, certain voids in the artery cannot reflect WSS variations in these spaces. Additionally, the manual positioning of aneurysm lesions introduces some rigidity. Future work will explore more flexible approaches to address these limitations.

## Figures and Tables

**Table 1:** Pre-defined parameters involved in our model of aneurysmal lesion.

Parameter	Value	Unit	Description
*h*	0.025	m	kernel radius
$\rho_{f}$	1000.0	${\mathrm{kg}/m}^{3}$	Density of tissue fluid
*c*	1500.0	m/s	Propagation velocity in the wave equation
*s*	0.01	$m^{2}$	Cross-sectional area of tissue fluid acting on aneurysm
$K_{C}$	3.52	N/m	Scale factor of collagen
$K_{E}$	800.0	N/m	Scale factor of elastin
$S_{C}$	10.0	$m^{2}$	Cross-sectional area of collagen
$S_{E}$	20.0	-	Cross-sectional area of elastin
μ	4.0	-	Viscosity coefficient of blood fluid
$\mu_{B}$	30.0	-	Viscous coefficient of internal forces in blood vessels
Δt	1.0	ms	Simulation time step

**Table 2:** Parameter design cases of bifurcating arteries with different structures in our experiment.

Case	Bifurcation Index (*n*)	Bifurcation Angle (*A*)	Radius Ratio (r)
G1	2	75.00°	1.0
G2	3	75.00°	1.0
G3	4	75.00°	1.0
G4	3	45.00°	1.0
G5	3	75.00°	1.0
G6	3	105.00°	1.0
G7	3	75.52°	0.4
G8	3	75.03°	0.7
G9	3	75.00°	1.0

**Table 3:** Efficiency of the proposed method under four groups of test conditions.

TC	Number of Particles	Aneurysm	FPS (CPU)	FPS (CUDA (CPU + GPU))
1	5520	✔	5.65	86.95
2	15,456	✔	1.61	54.56
3	15,456	×	1.72	54.15
4	40,296	✔	0.45	11.10

## Data Availability

The raw data supporting the conclusions of this article will be made available by the authors on request.

## Supplementary Materials

The following supporting information can be downloaded at: https://www.mdpi.com/article/10.3390/bioengineering11121200/s1, Video S1: Demonstration of Visual Simulation for Bifurcation Aneurysms and Wall Shear Stress (WSS) Distribution.
